# Contributing factors related to abnormal uterine bleeding in perimenopausal women: a case–control study

**DOI:** 10.1186/s41043-024-00540-4

**Published:** 2024-04-18

**Authors:** Yuan Tian, Bin Bai, Li Wang, Zongchang Zhou, Jiahui Tang

**Affiliations:** 1 Department of Obstetrics and Gynecology, Clinical Skill Experiment Center of Haiyuan College, Kunming Medical University, Kunming, China; 2 Department of Critical Medicine, The Third People’s Hospital of Kunming, Kunming, China; 3 Department of Gynaecology, Kunming Tongren Hospital, Kunming, China; 4 Department of Obstetrics and Gynecology, Department of Obstetrics and Gynecology, Shuifu People’s Hospital, 150 Meters West of the Intersection of Shanghai Road and Ping’an Road, Gaotan New District, Shuifu City, Zhaotong City, 657800 Yunnan Province China

**Keywords:** Abnormal uterine bleeding, Diagnosis, Influencing factors, Perimenopause, Treatment

## Abstract

Abnormal uterine bleeding (AUB) during the menopausal transition results in reproductive endocrine disorders and both physiological and pathological changes, substantially impacting women’s health. This study aimed to investigate the factors influencing AUB in perimenopausal women. Between April 2021 and June 2022, 120 perimenopausal women with AUB in the menopausal transition, diagnosed and treated at the Gynaecology Department of Kunming Tongren Hospital, were included in the case group. Concurrently, women undergoing routine health examinations at the same hospital were randomly selected as the control group. Univariate and multivariate logistic regression analyses identified factors related to AUB. The univariate analysis revealed significant associations (*P* < 0.05) between AUB and several factors, including age, body mass index (BMI), age at menarche, gravidity, and intrauterine device (IUD) placement in perimenopausal women. The multivariate regression analysis indicated that the independent risk factors for AUB include benign endometrial lesions (odds ratio [OR] 5.243, 95% confidence interval [CI] 3.082–9.458, *P* < 0.001), endometrial thickness ≥ 10 mm (OR 1.573, 95% CI 0.984–3.287, *P* < 0.001), age ≥ 50 years (OR 2.045, 95% CI 1.035–4.762, *P* = 0.001), BMI ≥ 25 kg/m^2^ (OR 2.436, 95% CI 1.43–4.86, *P* = 0.002), and IUD placement (OR 2.458, 95% CI 1.253–4.406, *P* < 0.001). Abnormal uterine bleeding during the menopausal transition is associated with several factors, including age, BMI, and IUD placement, highlighting the importance of early screening for these risk factors in the diagnosis and treatment of AUB.

## Introduction

The menopausal transition, a crucial phase in the progression from puberty to the end of reproductive life, substantially impacts middle-aged women’s lives. This transition typically begins around the age of 40 and occurs mainly between 41 and 55 years. During this period, 75–80% of women experience various physical and psychological symptoms, including abnormal uterine bleeding (AUB) and perimenopausal syndrome [1, 2]. Abnormal uterine bleeding is characterised by abnormal menstrual vaginal bleeding, extended menstrual flow, or abnormally increased menstrual flow. Numerous causes of AUB in perimenopausal women have been identified, such as intrauterine polyps (AUB-P), adenomyosis (AUB-A), and uterine leiomyoma (AUB-L). Notably, patients may suffer from one or more diseases causing or relating to AUB [3, 4].

Abnormal uterine bleeding has become a prevalent gynaecological issue. A 2001 survey in China found an AUB incidence of 34.5% among 9,951 women patients [5], and approximately 25% of women in the United States have undergone gynaecological surgery for this condition [6]. Menopausal transition AUB often lacks distinctive early symptoms, leading to frequent misdiagnosis or oversight. If untreated, AUB can lead to complications such as anaemia, infection, and even the risk of malignant transformation into endometrial carcinoma (EC) [7]. Abnormal uterine bleeding substantially affects women’s physical and mental health, impacting their quality of life and potentially endangering their lives. Therefore, investigating the factors influencing AUB is crucial for future prevention and carcinogenesis management.

Early, accurate examination and diagnosis are key to improving early diagnosis rates and timely, effective treatment in postmenopausal women with AUB [8]. Although the substantial impact of AUB on women’s health and quality of life is recognised, there is a knowledge gap regarding specific risk factors and early diagnostic methods. Current endometrial pathology diagnoses rely on non-invasive imaging modalities, such as sonography, computed tomography, and magnetic resonance imaging, and invasive procedures, such as blind biopsy and hysteroscopy [9]. However, these methods often only detect already abnormal diseased tissue and are limited in the early identification of individuals at high risk of developing AUB.

Consequently, this study investigates the various factors contributing to AUB during this pivotal phase. The study aims to assist clinicians in identifying high-risk factors for AUB during the menopausal transition, facilitating early diagnosis and effective treatment. Ultimately, this research may contribute to extending patients’ life expectancy, enhancing their quality of life, and promoting more effective disease management.

## Materials and methods

### Related definitions

The following criteria define abnormal uterine bleeding: ① a menstrual cycle length of < 21 days or > 35 days, with a bleeding duration exceeding 8–10 days; ② menstrual blood loss exceeding 80 ml per menstrual cycle, or a subjective feeling of excessive menstrual flow (some studies consider the soaking of six sanitary pads or tampons in a single day as abnormal); and ③ the presence of intermenstrual bleeding [10]. Since 2011, the International Federation of Gynecology and Obstetrics has classified the underlying causes of AUB into a system known as PALM–COEIN. Under PALM classification, various aetiologies involve structural modifications that can be identified using imaging methodologies or histopathological assessments. These include AUB-P, AUB-A, AUB-L (also referred to as fibroids), endometrial malignancies, and endometrial hyperplasia with atypia. On the other hand, the COEIN category encompasses conditions in which structural changes within the uterus are absent, including coagulopathy, ovulatory dysfunction, endometrial-related factors, iatrogenic influences, and unclassified causes. Additionally, within this classification, AUB-Ls are further subcategorised based on their location, being classified as either submucosal or situated in other areas [3].

Natural menopause is marked by the cessation of menstruation for a duration of 12 months and typically occurs between the ages of 49 and 52. However, the timing of menopause varies across different ethnic groups, with a median age of 51.4 years observed in high-income nations [11]. Perimenopause refers to the transitional phase in midlife that precedes the end of reproductive capability in women. Currently, over 850 million women worldwide are aged between 40 and 60 years, with 88% experiencing a perimenopausal transition. This transition typically occurs at an average age of 51.4 years, following a Gaussian distribution ranging from 40 to 58 years [12]. Premenopause is a relative term denoting the absence of symptoms associated with perimenopause or menopause.

### Research participants

This study involved 120 women undergoing menopausal transition and experiencing AUB. These participants, diagnosed and treated in the Department of Gynaecology at Kunming Tongren Hospital between April 2021 and June 2022, formed the case group. These women presented with varying degrees of menstrual cycle disorders, prolonged menstruation, increased menstrual flow, or mild anaemia. The inclusion criteria for this group included the following: ① aged between 41 and 55 years (menopausal transition period) [1]; ② exhibiting clinical manifestations of AUB; ③ having a definite pathological diagnosis; and ④ possessing complete case data. The exclusion criteria included the following: ① aged < 41 or > 55 years; ② bleeding from the cervix, external vagina, or urethra; ③ pregnancy; ④ coagulation disorders; ⑤ severe liver disease; ⑥ presence of other reproductive system tumours; and ⑦ incomplete patient data.

**Fig. 1:**
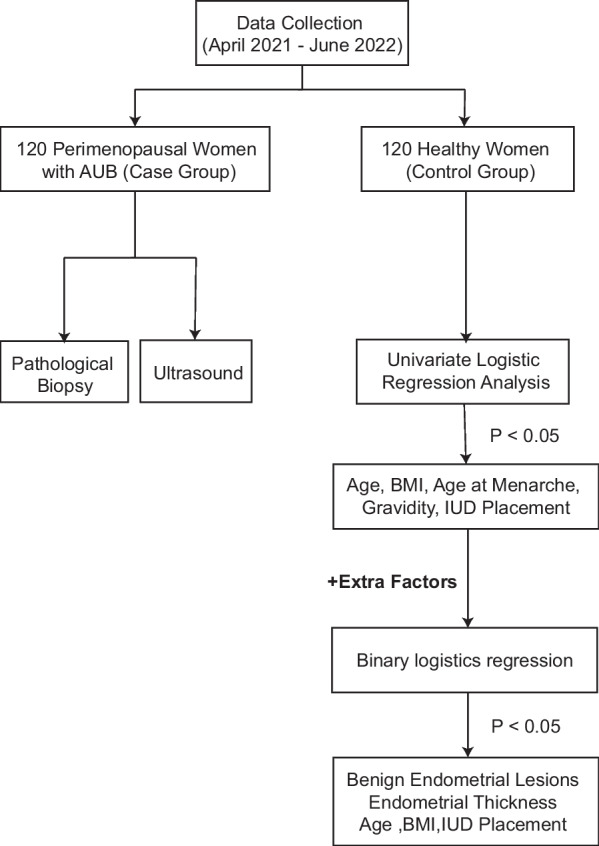
A flowchart covering the whole process of this work

The control group comprised another 120 healthy women who underwent physical examinations at the same hospital during the same period. The inclusion criteria for the control group included the following: ① aged between 41 and 55 years (menopausal transition period); and ② absence of AUB. The exclusion criteria included the following: ① presence of systemic diseases; ② presence of medical diseases; and ③ incomplete data. All participants were informed about the study and provided signed informed consent. A flowchart illustrating the entire process of this study is presented in Fig. 1.

The required sample size (N) for the study was calculated using logistic regression according to the following formula:

$$N=\frac{{Z}_{1-\alpha /2}^{2}*{\text{p}}(1-{\text{p}})}{{d}^{2}}$$

Here, the event occurrence (p) refers to the incidence of AUB [5, 6], set at 0.30 (30%). The significance level (α) was established at 0.05, with Z_1-α/2_ being the Z-score corresponding to the chosen significance level (α/2). For α = 0.05, Z_1-α/2_ is approximately 1.96. Effect sizes (*d*) typically range from small to medium, estimated between 0.2 and 0.5; here, we set *d* = 0.20. Based on these parameters, approximately 20 samples were required for subsequent analysis. Having 120 samples in each group (case and control) ensured that the statistical requirements for this study were met.

### Investigation methods

An investigation team was established, comprising the attending physician and a data collector. Printed survey questionnaires were distributed for face-to-face investigations. The survey included the following general clinical data: ① age; ② BMI; ③ age at menarche; ④ gravidity; ⑤ parity; ⑥ number of miscarriages; ⑦ whether an IUD was inserted; ⑧ whether menopause was delayed; ⑨ medical history, including hypertension, diabetes, hyperlipidaemia, thyroid disease, use of oestrogen drugs, and genital tract inflammatory diseases; and ⑩ family history. The classification of obesity in Asian women is BMI ≥ 25 kg/m^2^ [13–15].

### Examination method

After a gynaecological examination, procedures such as colour Doppler ultrasonography, hysteroscopy, and pathological biopsy of endometrial curettage were performed.

① Ultrasonography: This modality objectively reflects the size and shape of the uterus, endometrial thickness, endometrial echo, and endometrial blood flow signal, aiding in the identification of uterine and adnexal lesions. A colour Doppler ultrasound diagnostic instrument (MyLab™ 30Gold, Italy Esaote Medical Equipment Co.), with a CA1421 probe type and a probe frequency of 2.0–6.0 MHz, was employed. The operation was transabdominal. Image acquisition and data measurement were conducted by different physicians to ensure repeatability between the observers.
② Hysteroscopy: As a newer examination method, hysteroscopy has gained increasing favour among clinicians for its advantages of direct visibility and clear vision. It allows for a clear view of the internal structure of the entire uterine cavity, facilitating the selection of lesions under direct vision for biopsy. This is conducive to diagnosing various endometrial lesions, such as AUB-P, submucosal endometrial fibroids, and EC.
③ Endometrial pathological examination: Pathological biopsy of endometrial tissue taken by diagnostic curettage or hysteroscopy is the gold standard for the diagnosis of AUB.

The pathological changes of the endometrium were classified according to the World Health Organization’s 1994 classification. Endometrial hyperplasia is divided into four groups based on glandular complexity and nuclear atypia: non-atypical endometrial hyperplasia (simple, complex) and atypical endometrial hyperplasia (simple, complex) [16].

### Statistical analysis

This study used SPSS 26.0 software (IBM Corp, Chicago, IL, USA) for the statistical analysis. The normality of the quantitative data was assessed using the Kolmogorov–Smirnov test (test level: *P* = 0.05). Quantitative data conforming to a normal distribution were presented as means ± standard deviations ($\overline{ \times } \pm {\text{s}}$). An independent sample t-test was employed for comparisons between the groups. Data not normally distributed were expressed as the median (25th–75th percentile) and compared using the Mann–Whitney U test. Qualitative data were presented as n (%). The χ^2^ test was chosen if the total number of cases was > 40 and the theoretical frequency was > 5; otherwise, Fisher’s exact probability test was uniformly selected. Important factors, as determined by univariate analysis results and professional knowledge, were included. Binary logistic regression was used for the multivariate analysis to calculate the odds ratio (OR) and regression coefficient of each risk factor. A *P*-value of < 0.05 was considered statistically significant. Variable assignments for the logistic regression analysis were as follows: ① pathological outcomes: ‘1’ = normal endometrium; ‘2’ = benign changes of the endometrium, ‘3’ = EC or precancerous lesion; ② endometrial thickness: ‘1’ = endometrial thickness ≤ 10 mm, ‘2’ = endometrial thickness ≥ 10 mm; ③ endometrial echo: ‘1’ = uniform, ‘2’ = non-uniform; ④ age: ‘1’ =  < 50 years, ‘2’ =  > 50 years; ⑤ BMI: ‘1’ =  < 25 kg/m^2^, ‘2’ =  > 25 kg/m^2^; ⑥ age at menarche: ‘1’ =  < 12 years, ‘2’ =  > 12 years; ⑦ gravidity: ‘1’ =  < 1 time, ‘2’ =  > 1 time; ⑧ IUD placement: ‘1’ = ‘Yes’, ‘2’ = ‘No’; and ⑨ history: ‘1’ = ‘Yes’, ‘2’ = ‘No’.

## Results

### Comparison of pathological biopsy results between the two groups

**Fig. 2:**
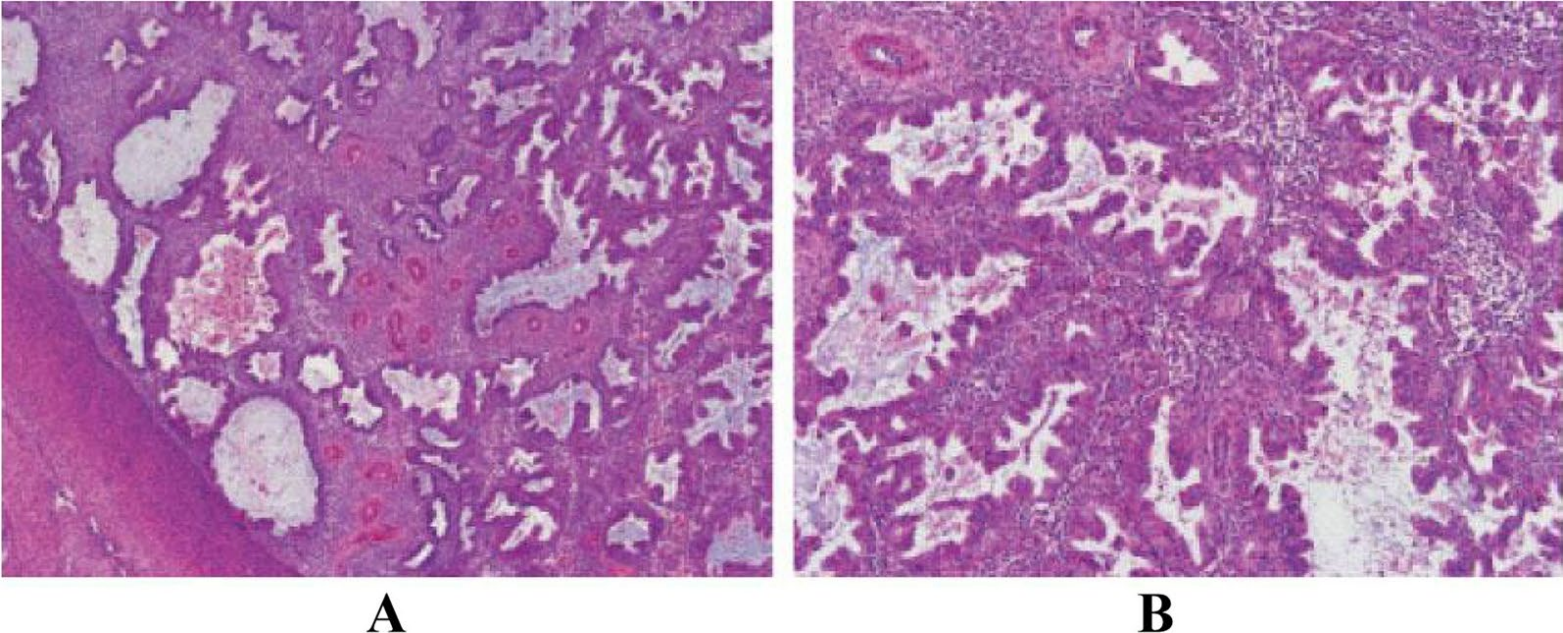
Pathological light microscopic features of endometriosis. **A** composed of hyperplastic endometrial glands and mesenchymal cells (HE × 40); **B** glands with complex hyperplasia and secretory changes (HE × 100)

The difference in the pathological biopsy results of the endometrium between the two groups was statistically significant (*P* < 0.001). The proportion of benign endometrial changes (68.3% vs. 9.2%) and cancer or precancerous changes (6.7% vs. 0.8%) was considerably higher in the case group compared with the control group, as detailed in Table 1 and illustrated in Fig. 2.

**Table 1:** Comparison of pathological results and ultrasonic examination results between two groups

Indicators	Case group (n = 120)	Control group (n = 120)	χ^2^	*P*
Pathological results			103.736	< 0.001
Normal endometrial	30 (25.0)	108 (90.0)		
Benign changes of endometrium	82 (68.3)	11 (9.2)		
Endometrial carcinoma or precancerous lesion	8 (6.7)	1 (0.8)		
Endometrial thickness (mm)			80.610	< 0.001
< 10	33 (27.5)	102 (85.0)		
≥ 10	87 (72.5)	18 (15.0)		
Endometrial echo			31.360	< 0.001
Uniform	54 (45.0)	96 (80.0)		
Non-uniform	66 (55.0)	24 (20.0)

### Comparison of ultrasound findings between the two groups

**Fig. 3:**
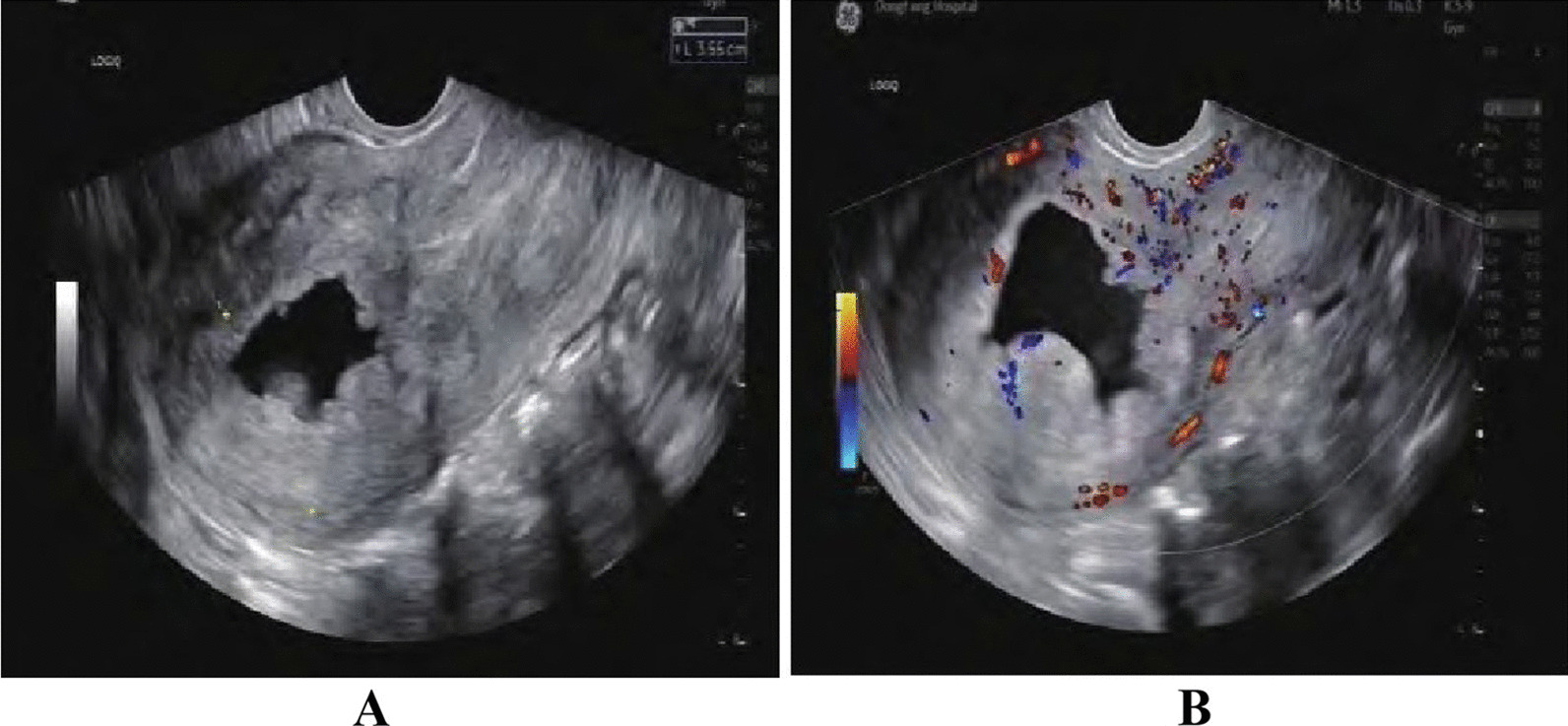
Imaging picture of a patient with endometrial cancer on transvaginal colour Doppler ultrasound. The patient was a female, 49 years old, with clinical presentation of irregular vaginal bleeding. The endometrial echogenicity was observed in 2D colour Doppler ultrasound, which showed that the endometrial echogenicity was heterogeneous and chaotic, with irregular endometrial morphology and contour, blurred echogenicity of the baseline, and unclear demarcation with the local myometrium, see **A**. Colour Doppler blood flow imaging showed rich blood flow signals, and the diagnosis of endometrial cancer was made, see **B**

The ultrasonography results of the case group were compared with those of the control group, focusing on endometrial thickness and endometrial echo. In line with the International Endometrial Tumor Analysis statement [17], the highest measurement in the sagittal plane for each patient, encompassing both endometrial layers (double endometrial thickness), was recorded as the endometrial thickness value. The proportion of women with an endometrial thickness of > 10 mm was markedly higher in the case group than in the control group, with a statistically significant difference (72.5% vs. 15%, *P* < 0.001). Similarly, the proportion of women with heterogeneous endometrial echogenicity was significantly higher in the case group than in the control group (55% vs. 20%, *P* < 0.001). These findings are shown in Table 1 and Fig. 3.

### Independent risk factors for abnormal uterine bleeding in perimenopausal women determined by univariate analysis

Univariate analysis revealed several notable differences between the case and control groups. The proportion of women aged > 50 years was higher in the case group (34.2% vs. 20%, *P* = 0.014). There were 37 obese women (BMI ≥ 25) in the case group, significantly more than in the control group (30.8% vs. 15.8%, *P* = 0.006). The proportion of women aged < 12 years at menarche in the case group was significantly higher than in the control group (15% vs. 5.8%, *P* = 0.020). The proportion of women with a gravidity of > 1 time in the case group was higher than that in the control group (65% vs. 35.8%, *P* < 0.001). Additionally, the proportion of IUD placement in the case group was significantly higher than that in the control group (76.7% vs. 63.3%, *P* = 0.024). No significant differences were found in terms of parity, the number of miscarriages, delayed menopause, medical history, and family history between the two groups (P > 0.05), as presented in Table 2.

**Table 2:** Single factor analysis of AUB in menopausal transition women

	Case group (*n* = 120)	Control group (*n* = 120)	*t/x*^2^	*P*
Age (year)			6.098	0.014
< 50	79 (65.8)	96 (80.0)		
≥ 50	41 (34.2)	24 (20.0)		
BMI (kg/m^2^)			7.547	0.006
< 25	83 (69.2)	101 (84.2)		
≥ 25	37 (30.8)	19 (15.8)		
Age at menarche (year)			5.403	0.020
≤ 12	18 (15.0)	7 (5.8)		
> 12	102 (85.0)	113 (94.2)		
Gravidity (time)			20.418	< 0.001
≤ 1	42 (35.0)	77 (64.2)		
> 1	78 (65.0)	43 (35.8)		
Parity (time)	1.695 ± 0.543	1.326 ± 0.652		0.819
Abortion (time)	0.431 ± 1.062	0.283 ± 0.945		0.073
IUD placement			5.079	0.024
Yes	92 (76.7)	76 (63.3)		
No	28 (23.3)	44 (36.7)		
Menopause delayed			0.603	0.438
Yes	59 (49.2)	53 (44.2)		
No	61 (50.8)	67 (55.8)		
Past history			1.068	0.301
Yes	62 (51.7)	54 (45.0)		
No	58 (48.3)	66 (55.0)		
Family history			2.017	0.156
Yes	2 (1.7)	0 (0.0)		
No	118 (98.3)	120 (100.0)		

### Independent risk factors for abnormal uterine bleeding in perimenopausal women determined by multivariate logistics regression analysis

Binary logistic regression was employed to incorporate factors with *P* < 0.05 from the results of the univariate analysis, as well as factors professionally considered to influence AUB, such as medical history, as independent variables. Abnormal uterine bleeding was utilised as the dependent variable, and stepwise regression was applied to identify relevant factors. To ensure the retention of explanatory variables in the model, the inclusion level was set at 0.10, and the exclusion level was set at 0.15.

**Fig. 4:**
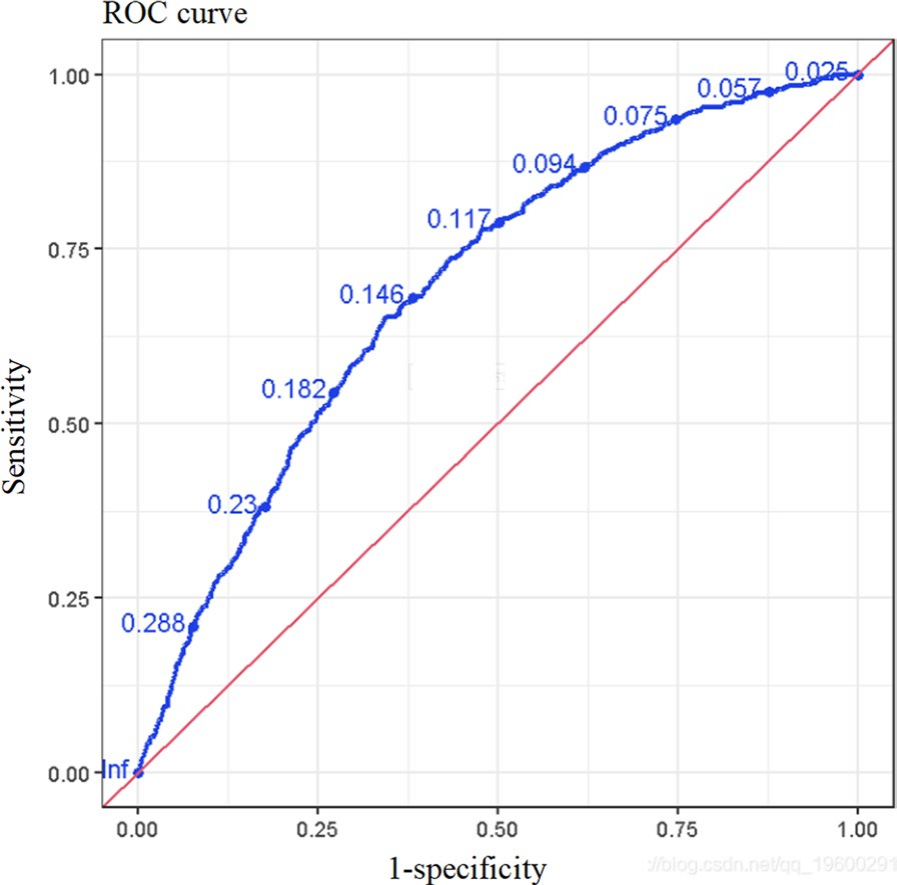
The ROC curve based on the multivariate logistics regression analysis

The logistic regression analysis results are presented in Table 3. Benign endometrial lesions (OR 5.243, 95% confidence interval [CI]: 3.082–9.458, *P* < 0.001), endometrial thickness ≥ 10 mm (OR 1.573, 95% CI 0.984–3.287, *P* < 0.001), age ≥ 50 years (OR 2.045, 95% CI 1.035–4.762, *P* = 0.001), BMI ≥ 25 kg/m^2^ (OR 2.436, 95% CI 1.143–4.986, *P* = 0.002), and IUD placement (OR 2.458, 95% CI 1.253–4.406, *P* < 0.001) were identified as independent risk factors for AUB in perimenopausal women. The ROC analysis result is shown in Fig. 4.

**Table 3:** Multivariate logistic regression analysis results of AUB in menopausal transition women

Variable	*β*	SE	Wald test	*P*	OR	95% CI	
						Upper Limit	Lower Limit
Pathologic outcomes (Benign changes of endometrium)	1.834	0.286	24.495	< 0.001*	5.243	3.082	9.458
Endometrial thickness (≥ 10)	0.852	0.365	6.463	< 0.001*	1.573	0.984	3.287
Endometrial echo (Non-uniform)	0.273	0.158	2.974	0.436	1.035	0.652	2.476
Age (≥ 50)	0.825	0.342	5.361	0.001*	2.045	1.035	4.762
BMI (≥ 25 kg/m^2^)	0.983	0.375	7.082	0.002*	2.436	1.143	4.986
Age at menarche (≤ 12 year)	−0.531	0.584	0.993	0.426	0.357	0.105	1.367
Gravidity (≤ 1time)	−0.324	0.487	0.521	0.382	0.735	0.281	1.843
IUD placement (No)	0.980	0.304	10.485	< 0.001*	2.458	1.253	4.406
Past history (No)	−0.593	0.524	1.438	0.350	0.645	0.284	1.525

## Discussion

Excessive menstrual bleeding, known as menorrhagia, has emerged as a prevalent manifestation of AUB in perimenopausal women. Alongside vaginal bleeding, other symptoms such as pallor, pelvic discomfort, and fatigue are frequently encountered. The duration of bleeding and the volume of blood loss prior to hospital admission are notably higher in perimenopausal individuals compared with their postmenopausal counterparts. Factors including age, menstrual characteristics, and diabetes heighten the susceptibility to endometrial cancer in women experiencing postmenopausal bleeding [18].

The results of this study indicated that the incidence of benign endometrial changes, cancer, or precancerous changes in women in the case group was notably higher than that in the control group. This suggests that benign endometrial changes or carcinogenesis are the primary causes of AUB. Moreover, due to dysfunction of the hypothalamic-pituitary-ovarian axis in perimenopausal women, the timing, amount, and ratio of oestrogen and progesterone production affecting the endometrium become imbalanced, leading to endometrial simple or complex hyperplasia [19]. Consequently, clinical practice should pay attention to physiological changes during the menopausal transition in women, and appropriate oestrogen supplementation to maintain certain physiological functions of the genital tract is beneficial in preventing infection.

In this study, colour Doppler ultrasound was employed to measure endometrial thickness and endometrial echogenicity. This technique allows for the visual detection of uterine size, intrauterine structure, the relationship of the endometrial lining, and blood flow distribution, offering the advantages of being non-invasive, allowing dynamic monitoring, and being repeatable [20]. The uterine artery Doppler indices, including resistance index, pulsatility index, and peak systolic velocity, exhibited substantially lower values in the malignant group than in the benign group. Endometrial thickness was found to be a reliable indicator for the diagnosis of endometrial cancer, achieving an area under the curve value of 0.89. For women with postmenopausal bleeding, a threshold value of 12.5 mm provided the highest levels of sensitivity and specificity [21]. The study also found that the proportion of women with endometrial thickness of > 10 mm was notably higher in the case group than in the control group, and the proportion of heterogeneous endometrial echogenicity was also higher. The potential reasons for this include the tendency of the endometrium in patients with AUB to show infiltrative growth, with benign changes or precancerous changes being more likely. Consequently, when lesions appear, they invade the endometrial myometrium and serosa, resulting in uneven endometrial echoes and unclear demarcation between the endometrium and myometrium [22]. It is suggested that colour Doppler ultrasonography can serve as a simple and effective tool for screening high-risk AUB groups in clinical practice by measuring endometrial thickness and echogenicity in the early evaluation of patients with AUB, offering substantial clinical application value.

The results of this study demonstrated that benign endometrial lesions, endometrial thickness ≥ 10 mm, age ≥ 50 years, BMI ≥ 25 kg/m^2^, and IUD placement are independent risk factors for AUB in perimenopausal women. These findings align with those reported by Xiao et al. [23], who identified IUD placement and obesity as risk factors for AUB in this demographic. A potential explanation for these results is that endometrial fibrinolytic activity increases following IUD placement, particularly in the early stages, when fibrinolytic activity is at its peak, leading to enhanced menstrual flow. Affected by IUDs, the endometrium augments prostaglandin production through its secretory effect, thereby increasing capillary permeability and fragility, resulting in a greater volume of menstrual blood [24]. Additionally, prolonged retention of the upper contraceptive ring in the uterine cavity may lead to inflammatory reactions and other forms of bleeding. Thus, it is advisable for perimenopausal women to remove the contraceptive device promptly to prevent postmenopausal bleeding and infection.

AUB may be an expression of hormonal milieu, or it could be the clinical presentation of benign or malignant lesions of female genital tract in perimenopausal woman. This study suggested that benign endometrial lesions was related to the risk for AUB in perimenopausal women. In line with our finding, Talukdar et al. [25] found that histopathology of endometrium in patients with AUB was predominantly simple typical type and that the majority of cases were diagnosed as fibroid uterus. Moreover, another study [26] demonstrated that structural causes of AUB were identified in 81.3% of cases, with adenomyosis (33.65%), concomitant adenomyosis and leiomyoma (31.5%), and leiomyoma (14.8%) being the most common. The present study also evinced that endometrial thickness ≥ 10 mm was an independent risk factor for AUB in perimenopausal women. Indeed, consistent with this result, a previous study [27] has showed that majority of women with AUB in aerimenopausal age had endometrial thickness of 10–12 mm (35.7%) followed by 7–9 mm (27.1%). Getpook et al. [28] concluded that endometrial thickness of 8 mm or less was less likely to be associated with malignant pathologies in premenopausal uterine bleeding. These findings indicated the significance of ultrasonography in measuring endometrial thickness and identifying organic causes in order to screen perimenopausal women at high risk for AUB.

Moreover, compared with women in other age groups, perimenopausal women tend to have a more stable lifestyle, better dietary conditions, lower activity levels, and more rest time. These factors, among others, contribute to the prevalence of menopausal transition obesity [29]. In this study, the proportion of obese women in the case group was considerably higher than in the control group, indicating that obesity is a risk factor for AUB. This condition may be attributed to the conversion of excessive androgens in the adipose tissue of obese women into estrone via aromatisation, leading to endometrial hyperplasia [30]. Therefore, managing body weight in perimenopausal women is crucial. Furthermore, this study underscores the importance of addressing obesity in clinical practice, as it facilitates the early detection and diagnosis of AUB in perimenopausal women.

Identifying and understanding the primary risk factors for AUB is crucial for the prevention and early detection of AUB in perimenopausal women. Investigating these influencing factors can assist healthcare professionals in identifying potential risks for AUB at an early stage. This facilitates the implementation of appropriate monitoring, examination, and intervention measures, thereby aiding in early intervention. Such steps enhance treatment success rates, reduce disease progression, and mitigate adverse outcomes. Consequently, the findings of this study are pivotal for improving the health of women in the perimenopausal period and providing essential guidance for clinical practice.

This study has several limitations. First, the study was conducted as a single-centre study in one hospital, which might limit the generalisability of the findings to a broader population. Second, the relatively short timeframe of the study, spanning between April 2021 and June 2022, may have influenced the comprehensiveness of the results. Furthermore, the study might not have accounted for all potential confounding variables that could affect the relationship between risk factors and AUB, and there may be additional clinical influences yet to be identified. It is also important to note that haemoglobin levels and uterine size are crucial indicators in assessing the nature of AUB. A related study observed a higher incidence of moderate to severe anaemia in perimenopausal women compared with that in postmenopausal women, and substantial differences in uterine size were noted between benign and malignant groups [31]. However, due to logistical constraints, collecting relevant data was not feasible in the current study. Future multicentre, large-sample studies that incorporate a wider range of potential influencing factors for AUB may help overcome these limitations.

## Conclusion

This study suggests that AUB in perimenopausal women may be influenced by several factors, such as benign endometrial lesions, an endometrial thickness of > 10 mm, an age of > 50 years, a BMI of > 25 kg/m^2^, and IUD placement. Early recognition of these potential influencing factors could provide crucial insights into the prevention of AUB in perimenopausal women.

## Data Availability

The datasets used and/or analysed during the current study are available from the corresponding author on reasonable request.
